# Supplementary material for: Step by Step through the Years—High vs. Low Energy Lead Extraction Using Advanced Extraction Techniques
Source: J Clin Med. 2022 Aug 19;11(16):4884. doi: 10.3390/jcm11164884 (PMC9410501; doi:10.3390/jcm11164884)
Supplement: Supplementary file 1 [file jcm-11-04884-s001.zip › jcm-1845362-supplementary.pdf]

Supplemental Table S1. Complete extraction success using various extraction devices, stratified by planned ICD lead extraction.

	Extraction Tools	Use	Complete Retrieval %	*p* Value
All patients	Evolution RL, Cook Medical	63.7%	93.0%	0.431
	SLS II, Cook Medical	29.5%	95.0%	
	TightRail, Spectranetics	6.7%	90.2%	
ICD group	Evolution RL, Cook Medical	64.7%	94.8%	0.353
	SLS II, Cook Medical	29.7%	97.0%	
	ThightRail, Spectranetics	5.5%	92.0%	
non-ICD group	Evolution RL, Cook Medical	60.8%	87.3%	1.000
	SLS II, Cook Medical	28.9%	88.9%	
	TightRail, Spectranetics	2.9%	87.5%	

ICD: implantable cardioverter defibrillator.

Supplemental Table S2. Temporal trend of procedural details in five-year intervals.

	Total	2001–2006	2006–2011	2011–2016	2016–2021	*p* Value
Extracted leads	1453	62	317	507	544	N/A
TLE procedures	667	30	139	237	261	N/A
age	70 (59–77)	62 (56–68)	67 (58–76)	70 (60–77)	71 (62–78)	*<0.001*
female gender	22.0%	23.3%	22.3%	21.1%	22.6%	0.977
**device details**						
device type						
PM	29.2%	30.0%	21.6%	30.8%	31.8%	*0.006*
ICD	34.8%	20.0%	30.9%	34.2%	39.1%	
CRT-P	2.2%	6.7%	1.4%	1.7%	2.7%	
CRT-D	33.7%	43.3%	46.0%	33.3%	26.4%	
Total leads	2.4 ± 0.8	2.4 ± 0.8	2.6 ± 1.0	2.5 ± 0.9	2.2 ± 0.7	*<0.001*
planned extraction of high energy lead	60.9%	56.7%	68.3%	56.1%	61.7%	0.118
Lead dwell time	67 (36–106)	44 (35–54)	56 (35–85)	73 (35–105)	79 (38–131)	*<0.001*
**Indications**						
Infection	55.0%	63.3%	62.6%	51.1%	53.6%	0.124
Lead dysfunction	39.4%	33.3%	30.9%	43.0%	41.4%	0.095
Other	6.4%	3.3%	6.5%	7.6%	5.7%	0.750
**Procedure**						
Procedure duration (min)	140 ± 60	138 ± 82	138 ± 68	143 ± 57	138 ± 56	0.679
Fluoroscopy time (min)	9.5 (5.2–17.2)	10.2 (5.0–26.6)	10.0 (5.0–17.5)	10.0 (5.3–17.7)	9.0 (5.5–16.9)	0.654
Fluoroscopy dose (Gy·cm²)	23 (10–51)	32 (19–54)	27 (13–67)	26 (12–54)	16 (7–40)	*<0.001*
Extracted leads	2.14 ± 0.96	2.07 ± 0.91	2.28 ± 1.10	2.13 ± 1.04	2.08 ± 0.81	0.339
Steps used						
Step 1	100%	100%	100%	100%	100%	1.000
Step 2	2.4%	0.0%	2.9%	5.1%	0.0%	*0.001*
Step 3	59.2%	60.0%	68.3%	46.8%	65.5%	*<0.001*
Step 4	11.8%	3.3%	14.4%	13.5%	10.0%	0.228
Step 5	0.3%	0%	0%	0%	0.8%	0.558
Complete retrieval						
Total	94.9%	96.7%	97.1%	94.1%	94.3%	0.569
Step 1	35.7%	40.0%	26.6%	44.7%	31.8%	*0.002*
Steps 1–2	37.3%	40.0%	28.8%	48.1%	31.8%	*<0.001*
Steps 1–3	84.6%	93.3%	84.9%	81.9%	85.8%	0.364
Steps 1–4	94.8%	96.7%	97.1%	94.1%	93.9%	0.528
Clinical success	99.0%	96.7%	99.3%	100.0%	98.1%	0.109
Any complication	4.0%	3.3%	7.2%	2.1%	4.2%	0.116
Intraprocedural	2.1%	3.3%	2.2%	1.3%	4.2%	

Postprocedural	1.9%	0.0%	5.0%	0.8%	1.5%

CRT: cardiac resynchronization therapy; ICD: implantable cardioverter defibrillator; PM: pacemaker; TLE: transvenous lead extraction.

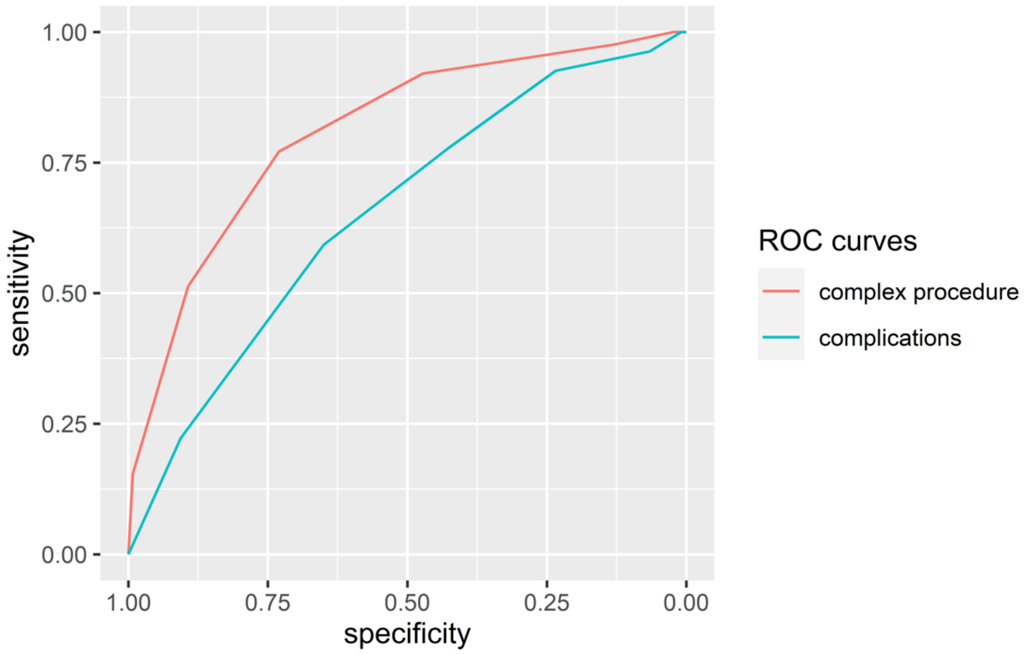

Supplemental Figure S1. ROC curves of MB score predicting the risk of a complex procedure (red, AUC 0.810) and short-term complications (turquoise, AUC 0.656).

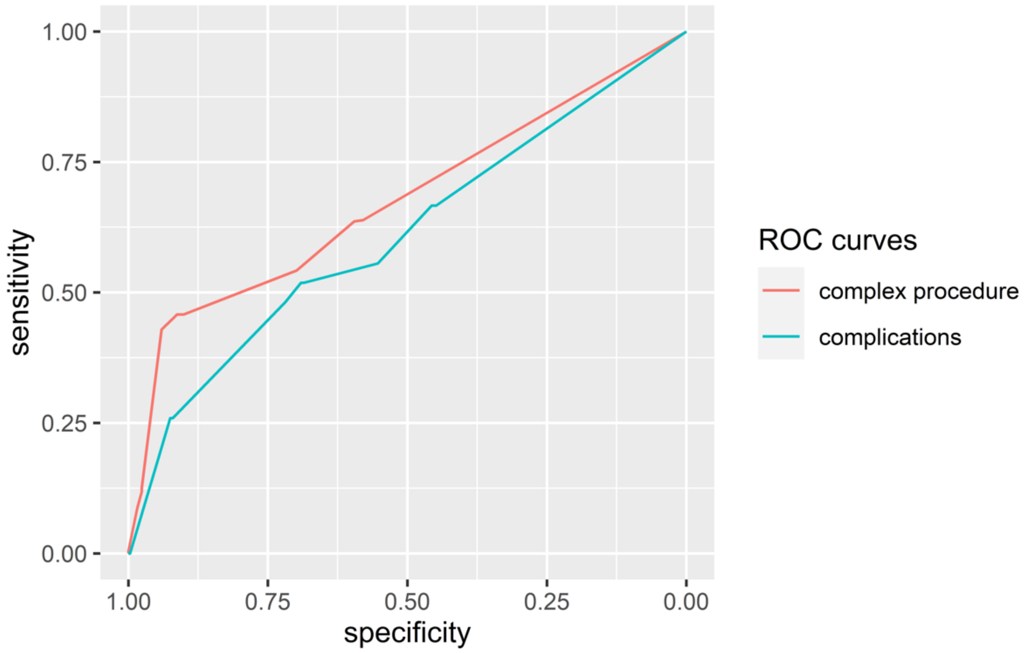

Supplemental Figure S2. ROC curves of modified SAFeTY TLE score predicting the risk of a complex procedure (red, AUC 0.676) and short-term complications (turquoise, AUC 0.611).
